# Potential Eligibility and Estimated Preventable Cardiovascular Disease Events From Inclisiran Treatment in the United States

**DOI:** 10.1016/j.jacadv.2025.102517

**Published:** 2026-01-20

**Authors:** Nathan D. Wong, Hridhay Karthikeyan, Wenjun Fan, Batul Electricwala

**Affiliations:** a Mary and Steve Wen Cardiovascular Division, University of California, Irvine School of Medicine, Irvine, California, USA; b Health Economics and Outcomes Research, Novartis Pharmaceuticals Corporation, East Hanover, New Jersey, USA

**Keywords:** cardiovascular disease, diabetes, inclisiran, prevention, statins

## Abstract

**Background:**

Many patients do not reach recommended low-density lipoprotein cholesterol (LDL-C) levels on statins alone. Inclisiran is a small interfering RNA providing ∼50% further reduction in LDL-C.

**Objectives:**

The authors estimated the number of U.S. adults eligible for inclisiran and the number of preventable atherosclerotic cardiovascular disease (ASCVD) events with inclisiran use.

**Methods:**

Using a cross-sectional study of the National Health and Nutrition Examination Survey 2011 to 2020, the authors identified 3 cohorts: high-risk primary prevention (≥20% 10-year ASCVD risk or 7.5% to <20% with ≥2 risk-enhancing factors), diabetes, and secondary prevention. Included adults were ≥18 years with LDL-C ≥70 mg/dL (≥55 mg/dL if very high-risk for ASCVD events) despite statin therapy. The ASCVD Pooled Cohort Equation and SMART2 risk score were used to estimate ASCVD events in 10 years “without inclisiran.” Preventable ASCVD events were calculated “with inclisiran” using inclisiran trial data to estimate LDL-C reduction and Cholesterol Treatment Trialists’ data to estimate reductions in ASCVD events per mmol/L of LDL-C.

**Results:**

Among the high-risk primary prevention, diabetes, and secondary prevention cohorts, the authors estimated 5.4 million, 6.5 million, and 8.0 million U.S. adults, respectively, to be eligible for inclisiran. Based on 34.5%, 29.0%, and 29.0% respective risk reductions, the authors estimated ∼448,600, ∼459,800, and ∼711,000 ASCVD events could be prevented over 10 years with inclisiran. Most preventable ASCVD events occurred among males and White patients.

**Conclusions:**

Approximately 20 million U.S. adults with inadequately controlled LDL-C may be eligible for inclisiran, with the potential to prevent over 1.6 million ASCVD events over 10 years.

Statin therapy is the foundation for lipid-mediated cardiovascular risk reduction; however, despite its widespread use, many patients do not reach their goal on statin therapy alone due to inadequate efficacy or suboptimal adherence,^1^ resulting in a significant residual atherosclerotic cardiovascular disease (ASCVD) risk.^2^ This residual risk highlights a significant opportunity for newer nonstatin therapeutic options, including bempedoic acid or therapies that target proprotein convertase subtilisin/kexin type 9 (PCSK9); these include monoclonal antibodies and inclisiran, a small interfering RNA therapy.^2^

Although the number of U.S. adults who could benefit from statin therapy is substantial, there are limited data regarding U.S. population eligibility for nonstatin therapies and the number of potential ASCVD events that could be prevented with the use of such therapies in eligible individuals. This information would be helpful to better understand the potential reduction in cardiovascular outcomes, disability, health care costs, and wider economic benefits of such therapies. One recent analysis among 2,729 U.S. individuals (representing 149.3 million U.S. adults), estimated that 65.8 million or 44.0% were eligible for statin therapy, but only 45% of those eligible were on statins. Overall, it was estimated that 9.7 million and 11.6 million adults could benefit from PCSK9i and icosapent ethyl, respectively, based on lipid profiles and existing therapies.^3^ However, this report did not factor in the lower recommended low-density lipoprotein cholesterol (LDL-C) threshold of 55 mg/dL that might require consideration of nonstatin therapies for those defined as very high-risk ASCVD.^4^ This high-risk category includes those who have had multiple ASCVD events or those with a single ASCVD event with multiple high risk conditions.^5^ In a Swiss cohort of patients with acute coronary syndrome, it was estimated that 51% of patients would be eligible for PCSK9i treatment based on European Society of Cardiology/European Atherosclerosis Society criteria and 14% according to American College of Cardiology (ACC)/American Heart Association (AHA) criteria.^6^ Moreover, a previous analysis from our team estimated that 3.0 million U.S. adults met REDUCE-IT (Reduction of Cardiovascular Events With Icosapent Ethyl–Intervention Trial) eligibility criteria for icosapent ethyl, with an estimated 71,391 cardiovascular disease (CVD) events that could be prevented annually.^7^

Inclisiran is a small interfering RNA therapy that inhibits the production of the PCSK9 protein and provides an approximate 50% reduction of LDL-C from injections at baseline, 3 months, and every 6 months thereafter.^8^ Cardiovascular outcomes trials are ongoing for both secondary prevention^9^^,^^10^ and high-risk primary prevention^11^ patients, but exploratory data from short-term follow-up of patients with ASCVD in phase III trials suggest ∼25% lower risk of future ASCVD events.^12^ Currently, estimates regarding the number of U.S. adults who may be potential candidates for inclisiran treatment, including those with pre-existing ASCVD, diabetes, or patients with multiple risk factors without ASCVD, are unavailable. Consequently, there is an opportunity to assess the potential number of ASCVD events that could be prevented with inclisiran use.

In this study, we estimated the number of U.S. adults potentially eligible for inclisiran therapy across 3 groups (secondary prevention, diabetes, and high-risk primary prevention) and, using anticipated event rates and relative risk reductions observed in previous LDL-C-lowering trials, we projected the number of ASCVD events that could potentially be prevented through inclisiran treatment.

## Methods

We conducted a cross-sectional study including participants aged 18 years and over from the U.S. National Health and Nutrition Examination Survey (NHANES), 2011 to 2020, who would be potentially eligible for inclisiran. NHANES is one of the largest and most comprehensive nationally representative cohorts of adults in the United States, comprising medical history, laboratory results, and medication information. The contemporary NHANES has been conducted continuously across multiple sites among representative samples of the U.S. population (both children and adults) since 1999, with data released every 2 years.^13^ Survey data are collected by questionnaire (including medical history of comorbidities and prescription data), and blood and urine samples are provided for laboratory measures. The NHANES surveys form the basis for U.S. population statistics on the prevalence of CVD, its components, and risk factors (prevalence and distribution), using population weighting measures applied to the sample estimates of prevalence and distribution of CVD. All NHANES participants provided informed consent. This analysis used deidentified publicly available data and therefore was exempt from Institutional Review Board review.

Three patient cohorts were identified in the analysis, and NHANES sample weighting procedures were used to project each sample to the general U.S. population (in millions).

### Secondary prevention population

We included individuals with known ASCVD based on self-report of prior heart attack, stroke, or coronary heart disease, who were on current statin therapy, and with an LDL-C of ≥70 mg/dL. The very high-risk ASCVD group was based on 2 major ASCVD events (prior heart attack or stroke) or 1 major ASCVD event and 2 or more high-risk conditions as available in NHANES (age ≥65 years, diabetes, hypertension, chronic kidney disease, currently smoking, elevated LDL-C, and history of congestive heart failure), in accordance with the 2018 Multisociety Cholesterol Management Guideline.^14^ For patients with ASCVD and diabetes, we included those with an LDL-C of 55 mg/dL or higher based on recent recommendations.^4^^,^^5^^,^^15^ This is a modification of the ongoing VICTORION-2 PREVENT trial inclusion criteria that incorporates the lower LDL-C threshold of 55 mg/dL for those with a very high-risk ASCVD.^8^

### High-risk primary prevention population

We included persons without known ASCVD and without diabetes, who were on statin therapy and considered high-risk primary prevention (based on the 10-year Pooled Cohort Equation [PCE] ASCVD risk of ≥20% or 7.5% to <20% plus 2 or more risk-enhancing factors) based on the inclusion criteria from the ongoing VICTORION-1 PREVENT trial.^11^ Risk-enhancing factors were based on the ACC/AHA/Multisociety cholesterol management guideline,^14^ including a family history of premature ASCVD (close relative with heart attack), primary hypercholesterolemia (LDL-C between 160 and 189 mg/dL or nonhigh-density lipoprotein cholesterol [HDL-C] between 190 and 219 mg/dL), metabolic syndrome, chronic kidney disease (estimated glomerular filtration rate <60 mL/min/1.73 m^2^), chronic inflammatory conditions (HIV, rheumatoid arthritis, and psoriasis), premature menopause (age<40 years), gestational diabetes, Black race (in lieu of South Asian, which was not available in the data), primary hypertriglyceridemia (triglycerides ≥175 mg/dL), and where available, elevated high-sensitivity C-reactive protein (≥2.0 mg/L) or apolipoprotein B (≥130 mg/dL). Metabolic syndrome was defined as 3 or more of the following: waist circumference ≥90 cm for males or ≥80 cm for females, triglycerides ≥150 mg/dL, blood pressure ≥130/85 mm Hg or on treatment for hypertension, fasting glucose 100 to 129 mg/dL, and HDL-C <40 mg/dL for males or <50 mg/dL for females.

### Diabetes population (without prior ASCVD)

We included patients with diabetes who had an LDL-C of ≥70 mg/dL despite statin therapy. Diabetes was defined based on the self-report of physician diagnosis of diabetes, glycated hemoglobin A1c (HbA1c) ≥6.5%, glucose of ≥126 mg/dL (fasting) or ≥200 mg/dL (nonfasting), or use of insulin or hypoglycemic medication as previously defined in NHANES.^16^

### Demographic and risk factor measures

We included information on demographic characteristics including age, sex, race/ethnicity (Asian, Black, Hispanic, White, other), blood pressure, total cholesterol, LDL-C, HDL-C, triglycerides, cholesterol-lowering treatment (statin, ezetimibe, niacin, fibrates, bile acid resins, and PCSK9i based on data through 2020), hypertension, and smoking status (current, past, and never). No patients had taken inclisiran as it was not available in routine clinical practice during the survey period; inclisiran was first approved by the Food and Drug Administration (FDA) in 2021.

### Estimation of ASCVD risk and preventable events

For the estimation of the baseline risk and expected number of ASCVD events in 10 years in the secondary prevention population, we estimated the 10-year risk of recurrent ASCVD events for each individual using the SMART2 risk score for persons with vascular disease as previously described^17^ (Supplemental Methods; recalibrated to North American populations). For the estimation of the baseline risk and expected number of ASCVD events in 10 years in the high-risk primary prevention and diabetes populations, we estimated the 10-year risk of initial ASCVD events using the PCE ASCVD risk score (Supplemental Methods).^18^ The mean risk was then multiplied by the unweighted and weighted population sizes for each of the 3 cohorts as described previously to obtain the estimated number of ASCVD events “without inclisiran.”

The expected number of ASCVD events in 10 years “with inclisiran” was estimated by multiplying sample sizes of our projected eligible secondary prevention, high-risk primary prevention, and diabetes cohorts by the respective mean 10-year ASCVD risks “with inclisiran” which were derived from multiplying our baseline risks by the HRs for ASCVD events we projected for these cohorts (0.71, 0.655, and 0.71, respectively). These HRs were estimated from extrapolating (multiplying) the ASCVD relative risk reductions obtained from the Cholesterol Treatment Trialists (CTT) Collaboration estimates per mmol/L reduction in LDL-C (21%, 25%, and 21% for secondary prevention, primary prevention, and diabetes cohorts, respectively) obtained from their meta-analysis of these study populations^19^^,^^20^ with the achieved LDL-C reductions (1.38 mmol/L or 53 mg/dL) from phase III clinical trials of inclisiran.^8^ This would translate to 29.0%, 34.5%, and 29.0% relative risk reductions (estimated HRs of 0.710, 0.655, and 0.710, respectively) from the LDL-C reductions observed from inclisiran.

The number of potentially preventable ASCVD events in 10 years was calculated as the difference between the number of estimated ASCVD events “without” and “with” inclisiran use as described previously. We also presented our estimates separately by sex, race/ethnicity, and age category among the diverse NHANES cohort. We further stratified data in the secondary prevention population according to ASCVD risk (very high risk or not very high risk) and in the high-risk primary prevention population according to inclusion criteria (≥20% 10-year risk, or 7.5% to <20% 10-year risk with 2 or more risk-enhancing factors). We have used similar methodology applying inclusion criteria from clinical trials for estimating eligibility and preventable ASCVD events for other diabetes and CVD therapies.^7^^,^^21^^,^^22^

## Results

### Sample selection and demographics

**Figure 1:**
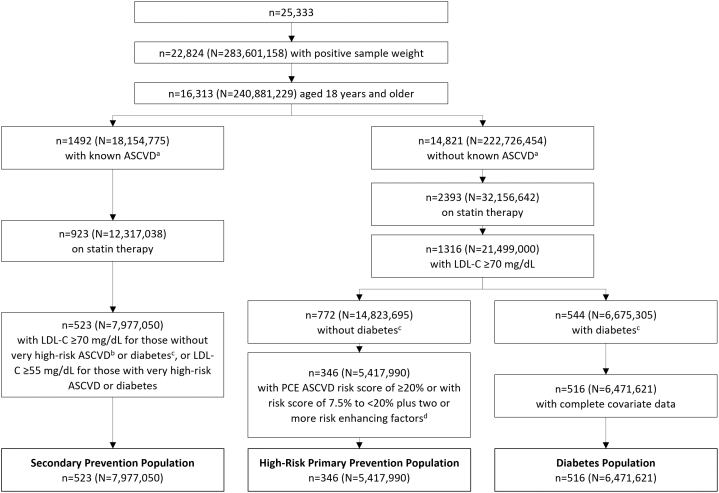
**Population Selection From the National Health and Nutrition Examination Survey database, 2011 to 2020** ^a^ASCVD was defined as prior self-reported diagnosis of coronary heart disease, heart attack, or stroke. ^b^Very high-risk ASCVD was defined as 2 major events, or 1 major event with ≥2 high-risk conditions. ^c^Diabetes was defined as self-reported physician diagnosis, glycated hemoglobin A1c ≥6.5%, glucose ≥126 mg/dL (fasting) or ≥200 mg/dL (nonfasting), or use of insulin or hypoglycemic medication. ^d^Risk-enhancing factors were family history of premature ASCVD, primary hypercholesterolemia, chronic kidney disease, chronic inflammatory conditions, premature menopause, gestational diabetes, Black race, primary hypertriglyceridemia, elevated high-sensitivity C-reactive protein, elevated apolipoprotein B, and metabolic syndrome defined ≥3 of increased waist circumference, high triglycerides, high blood pressure, elevated fasting glucose, and low high-density lipoprotein cholesterol, as defined in the methods. ASCVD = atherosclerotic cardiovascular disease; LDL-C = low-density lipoprotein cholesterol; n = population from NHANES; N = projected U.S. population; NHANES = national health and nutrition examination survey; PCE = pooled cohort equation.

Among 25,333 NHANES 2011 to 2020 participants, we identified 523 (projected to ∼8.0 million in the general U.S. population) who fit the eligibility criteria for inclisiran in the secondary prevention population, 346 (∼5.4 million) who fit the eligibility criteria for inclisiran in high-risk primary prevention population criteria, and 516 (∼6.5 million) who met the diabetes population criteria for a total of ∼20 million eligible U.S. adults (Figure 1, Central Illustration). Descriptive statistics on demographic and risk factor characteristics for each population are shown in Table 1. Between populations, mean age was slightly lower, and the proportion of Hispanic patients was higher in the diabetes population compared with the other 2 populations. CVD risk factors, such as total cholesterol, LDL-C, and triglycerides, were lowest in the secondary prevention population compared with the others. The secondary prevention population had nearly double the proportion of current smokers compared with the other populations. In all populations, use of statins was required, but usage of other cholesterol-lowering therapies was low (<10%).

**Central Illustration:**
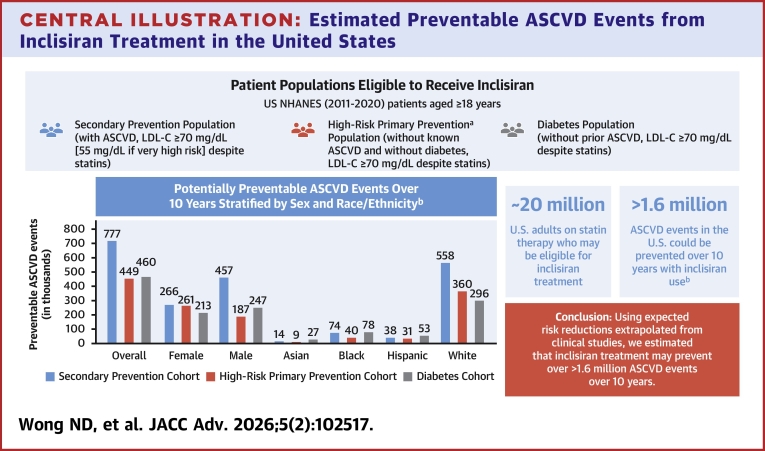
**Estimated Preventable ASCVD Events from Inclisiran Treatment in the United States** This cross-sectional analysis of NHANES 2011 to 2020 identified 3 cohorts: secondary prevention, high-risk primary prevention, and diabetes. It was estimated that ∼711,000, ∼448,600, and ∼459,800 ASCVD events could be prevented over 10 years with inclisiran treatment, respectively. Most preventable events occurred among males and White patients. Approximately 20 million U.S. adults with inadequately controlled LDL-C may be eligible for inclisiran, with the potential to prevent over 1.6 million ASCVD events over 10 years. ^a^Criteria for inclusion used a 10-year PCE ASCVD risk score of ≥20% or 7.5% to <20% plus 2 or more risk-enhancing factors based on the 2018 ACC/AHA/Multisociety cholesterol management guidelines. ^b^Risk reduction assumed LDL-C reductions for inclisiran were estimated at 53 mg/dL and assumes patients are eligible. ASCVD = atherosclerotic cardiovascular disease; LDL-C = low-density lipoprotein cholesterol; NHANES = national health and nutrition examination survey; PCE = pooled cohort equation.

**Table 1:** Descriptive Statistics Among Inclisiran Eligible Populations (From NHANES 2011-2020)

	Secondary Prevention Population (n = 523^a^) (8.0 million^b^)	High-Risk Primary Prevention Population (n = 346^a^) (5.4 million^b^)	Diabetes Population (n = 516^a^) (6.5 million^b^)
	Mean ± SEM	Mean ± SEM	Mean ± SEM
Age, y	67.1 ± 0.8	69.8 ± 0.7	62.0 ± 0.6
<65	173 (37.1, 3.0)	76 (25.9, 1.4)	278 (59.5, 3.9)
≥65	350 (62.9, 5.0)	270 (74.1, 4.0)	238 (40.5, 2.6)
Female	194 (37.7, 3.0)	171 (54.7, 3.0)	275 (53.0, 3.4)
Race or ethnic group			
White	278 (75.9, 6.1)	184 (79.2, 4.3)	157 (61.5, 4.0)
Asian	32 (2.5, 0.2)	15 (2.0, 0.1)	56 (6.0, 0.4)
Black or African American	112 (8.9, 0.7)	83 (11.0, 0.6)	154 (16.1, 1.0)
Hispanic or Latino	80 (5.6, 0.4)	56 (6.2, 0.3)	134 (13.8, 0.9)
Other	21 (7.1, 0.6)	8 (1.6, 0.1)	15 (2.7, 0.2)
Blood pressure, mm Hg			
Systolic	129.1 ± 1.3	134.0 ± 1.3	129.7 ± 0.9
Diastolic	78.2 ± 1.8	67.7 ± 1.1	81.0 ± 1.5
Total cholesterol, mg/dL	169.5 ± 1.5	191.1 ± 2.4	175.6 ± 2.2
Low-density lipoprotein cholesterol, mg/dL	93.1 ± 1.4	109.0 ± 2.2	98.1 ± 1.5
High-density lipoprotein cholesterol, mg/dL	51.0 ± 1.4	54.6 ± 1.1	51.1 ± 0.8
Triglycerides, mg/dL	127.2 ± 4.7	137.7 ± 4.7	131.9 ± 3.9
Cholesterol-lowering treatment			
Statin	523 (100, 8.0)	346 (100, 5.4)	516 (100, 6.5)
Ezetimibe	13 (3.0, 0.2)	7 (2.5, 0.1)	11 (3.3, 0.2)
Niacin	9 (3.3, 0.3)	1 (0.4, <0.1)	3 (0.9, 0.1)
Fibrates	17 (6.5, 0.5)	1 (1.0, 0.1)	12 (2.2, 0.1)
Bile acid resins	0	2 (2.1, 0.1)	0
PCSK9 inhibitors (monoclonal antibodies)	0	0	0
Has diabetes	261 (43.9, 3.5)	0	516 (100, 6.5)
Has hypertension	458 (81.0, 6.5)	280 (81.0, 4.4)	447 (82.1, 5.3)
Smoking status			
Current	110 (23.4, 1.9)	52 (13.5, 0.7)	63 (12.3, 0.8)
Past	220 (43.2, 3.4)	147 (47.1, 2.6)	180 (35.2, 2.3)
Never	193 (33.4, 2.7)	147 (39.4, 2.1)	273 (52.5, 3.4)

Values are mean ± SEM, or n (%, million).

NHANES = national health and nutrition examination survey; PCSK9 = proprotein convertase subtilisin/kexin type 9.

a Number referring to the patient population from the NHANES database.

b Million referring to the population estimate projected from sample size.

### Changes in CVD 10-year risk and preventable ASCVD events

**Figure 2:**
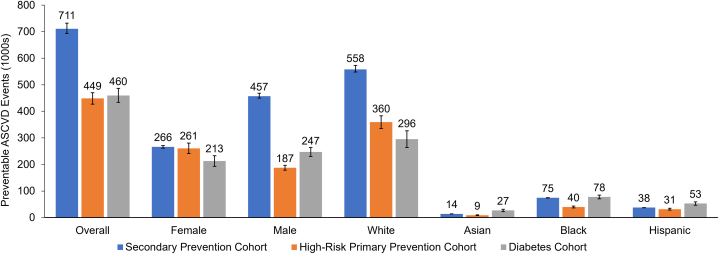
**Preventable Atherosclerotic Cardiovascular Disease Events Over 10 Years** Error bars show 95% CIs. ASCVD = atherosclerotic cardiovascular disease.

Ten-year CVD risk (%) estimates without and then with inclisiran treatment in our study sample are shown in Table 2, 3, and 4 along with the corresponding numbers of estimated ASCVD events, with the difference being the preventable ASCVD events. An overview of estimated preventable ASCVD events over 10 years is presented in Figure 2 and the Central Illustration.

**Table 2:** Estimated Cardiovascular Events and Preventable Events in Secondary Prevention Population, Preinclisiran and Postinclisiran Treatment, Based on SMART2 Risk Score

	n (N)	10-Year CVD Risk Pretreatment, % (95% CI)	10-Year CVD Risk Post-Treatment, % (95% CI)^a^	Difference, % (95% CI)	CVD Events Pre n (95% CI)	CVD Events Post n (95% CI)	Difference (Preventable Events) n (95% CI)
					K (95% CI)	K (95% CI)	K (95% CI)
Overall	523 (7,977,050)	30.7 (30.0-31.7)	21.8 (21.3-22.5)	8.9 (8.7-9.2)	160.7 (156.8-165.5)	114.1 (111.3-117.5)	46.6 (45.5-48.0)
					2,451.7 (2,391.5-2,524.7)	1,740.7 (1,698.0-1,792.6)	711.0 (693.5-732.2)
Females	194 (2,999,170)	30.6 (30.1-31.2)	21.7 (21.4-22.1)	8.9 (8.7-9.0)	59.4 (58.4-60.5)	42.2 (41.4-42.9)	17.2 (16.9-17.5)
					918.6 (902.2-935.1)	652.2 (640.5-664.0)	266.4 (261.6-271.2)
Males	329 (4,977,880)	31.7 (31.1-32.4)	22.5 (22.1-23.0)	9.2 (9.0-9.4)	104.1 (102.5-106.6)	73.9 (72.7-75.7)	30.2 (29.7-30.9)
					1,575.7 (1,550.1-1613.3)	1,118.8 (1,100.6-1,145.5)	457.0 (449.5-467.9)
White	278 (6,055,007)	31.8 (31.2-32.6)	22.6 (22.2-23.2)	9.2 (9.1-9.5)	88.4 (86.8-90.7)	62.7 (61.6-64.4)	25.6 (25.2-26.3)
					1,924.5 (1,891.0-1,975.1)	1,366.4 (1,342.6-1,402.4)	558.1 (548.4-572.8)
Asian	32 (195,869)	24.8 (24.7-25.0)	17.6 (17.5-17.7)	7.2 (7.2-7.2)	7.9 (7.9-8.0)	5.6 (5.6-5.7)	2.3 (2.3-2.3)
					48.6 (48.4-48.9)	34.5 (34.3-34.7)	14.1 (14.0-14.2)
Black	112 (713,203)	36.0 (35.7-36.3)	25.6 (25.3-25.8)	10.4 (10.3-10.5)	40.3 (40.0-40.7)	28.6 (28.4-28.9)	11.7 (11.6-11.8)
					256.7 (254.5-259.0)	182.3 (180.7-183.9)	74.5 (73.8-75.1)
Hispanic	80 (447,842)	29.0 (28.8-29.2)	20.6 (20.4-20.7)	8.4 (8.3-8.5)	23.2 (23.0-23.3)	16.5 (16.4-16.6)	6.7 (6.7-6.8)
					129.8 (128.9-130.6)	92.1 (91.5-92.7)	37.6 (37.4-37.9)
Age <65 y	173 (2,955,735)	22.6 (22.3-22.9)	16.0 (15.8-16.2)	6.5 (6.45-6.64)	39.0 (38.5-39.6)	27.7 (27.3-28.1)	11.3 (11.2-11.5)
					667.0 (657.7-676.3)	473.6 (466.9-480.2)	193.4 (190.7-196.1)
Age ≥65 y	350 (5,021,315)	36.2 (35.6-37.0)	25.7 (25.3-26.3)	10.5 (10.3-10.7)	126.6 (124.6-129.5)	89.9 (88.5-92.0)	36.7 (36.1-37.6)
					1,815.9 (1,788.1-1,858.4)	1,289.3 (1,269.5-1,319.5)	526.6 (518.5-538.9)
VHR ASCVD with LDL-C ≥55 mg/dL	405 (5,924,933)	31.4 (30.7-32.2)	22.3 (21.8-22.8)	9.1 (8.9-9.3)	127.3 (124.4-130.2)	90.4 (88.3-92.5)	36.9 (36.1-37.8)
					1,862.8 (1,819.5-1,905.5)	1,322.6 (1,291.9-1,352.9)	540.2 (527.7-552.6)
Not VHR ASCVD with LDL-C ≥70 mg/dL	118 (2,052,117)	30.7 (30.3-31.1)	21.8 (21.5-22.1)	8.9 (8.8-9.0)	36.3 (35.8-36.7)	25.7 (25.4-26.1)	10.5 (10.4-10.7)
					630.6 (622.2-639.0)	447.7 (441.8-453.7)	182.9 (180.4-185.3)

Estimates combining strata may not total overall due to rounding error. Those of other races, n = 21 (565,128), are not shown.

ASCVD = atherosclerotic cardiovascular disease; CVD = cardiovascular disease; K = number, referring to the projected U.S. patient population in 1000s; LDL-C = low-density lipoprotein cholesterol; N = number, referring to the projected U.S. patient population; VHR = very high-risk; other abbreviations as in Table 1.

a Post-treatment values calculated by multiplying pretreatment values by 0.71.

**Table 3:** Estimated Cardiovascular Events and Preventable Events in High-Risk Primary Prevention Population, Preinclisiran and Postinclisiran Treatment, Based on Pooled Cohort Equation ASCVD Risk Score

	n (N)	10-Year CVD Risk Pretreatment, % (95% CI)	10-Year CVD Risk Post-Treatment, % (95% CI)^a^	Difference, % (95% CI)	CVD Events Pre n (95% CI)	CVD Events Post n (95% CI)	Difference (Preventable Events) n (95% CI)
					K (95% CI)	K (95% CI)	K (95% CI)
Overall	346 (5,417,990)	24.0 (22.9-25.1)	15.7 (15.0-16.5)	8.3 (7.9-8.7)	83.0 (79.1-87.0)	54.4 (51.8-57.0)	28.6 (27.3-30.0)
					1,300.3 (1,238.2-1,362.2)	851.7 (811.0-892.3)	448.6 (427.2-470.0)
Females	171 (2,961,450)	25.5 (23.6-27.5)	16.7 (15.5-18.0)	8.8 (8.1-9.5)	43.6 (40.4-47.0)	28.6 (26.4-30.8)	15.0 (13.9-16.2)
					755.2 (699.1-813.6)	494.6 (457.9-532.9)	260.5 (241.2-280.7)
Males	175 (2,456,539)	22.1 (21.0-23.3)	14.5 (13.8-15.2)	7.6 (7.3-8.0)	38.7 (36.8-40.7)	25.3 (24.1-26.7)	13.3 (12.7-14.0)
					542.9 (516.3-571.4)	355.6 (338.2-374.3)	187.3 (178.1-197.1)
White	184 (4,289,990)	24.3 (22.7-25.9)	15.9 (14.9-17.0)	8.4 (7.8-8.9)	44.7 (41.7-47.7)	29.3 (27.3-31.2)	15.4 (14.4-16.4)
					1,042.5 (973.0-1111.6)	682.8 (637.3-728.1)	359.7 (335.7-383.5)
Asian	15 (108,495)	23.6 (20.1-27.1)	15.5 (13.2-17.8)	8.1 (6.9-9.4)	3.5 (3.0-4.1)	2.3 (2.0-2.7)	1.2 (1.0-1.4)
					25.6 (21.8-29.4)	16.8 (14.3-19.3)	8.8 (7.5-10.1)
Black	83 (597,582)	19.3 (17.6-21.0)	12.6 (11.5-13.8)	6.7 (6.1-7.3)	16.0 (14.6-17.5)	10.5 (9.6-11.4)	5.5 (5.0-6.0)
					115.3 (105.1-125.8)	75.5 (68.8-82.4)	39.8 (36.2-43.4)
Hispanic	56 (335,976)	27.1 (24.1-30.0)	17.8 (15.8-19.7)	9.3 (8.3-10.4)	15.2 (13.5-16.8)	9.9 (8.8-11.0)	5.2 (4.7-5.8)
					91.0 (81.0-100.9)	59.6 (53.1-66.1)	31.4 (27.9-34.8)
Age <65 y	76 (1,403,908)	12.4 (11.6-13.2)	8.1 (7.6-8.7)	4.3 (4.0-4.6)	9.4 (8.8-10.0)	6.2 (5.8-6.6)	3.3 (3.0-3.5)
					174.1 (162.4-185.6)	114.0 (106.4-121.6)	60.1 (56.0-64.0)
Age ≥65 y	270 (4,014,082)	28.1 (26.8-29.3)	18.4 (17.6-19.2)	9.7 (9.2-10.1)	75.9 (72.4-79.1)	49.7 (47.4-51.8)	26.2 (25.0-27.3)
					1,128.0 (1,076.1-1,176.4)	738.8 (704.9-770.5)	389.1 (371.3-405.9)
≥20% 10-year ASCVD risk	224 (3,099,489)	32.6 (31.4-33.7)	21.3 (20.6-22.1)	11.2 (10.8-11.6)	73.0 (70.4-75.6)	47.8 (46.1-49.5)	25.2 (24.3-26.1)
					1,010.1 (974.3-1,045.8)	661.6 (638.2-685.0)	348.5 (336.1-360.8)
7.5% to <20% 10-year ASCVD risk with ≥2 risk-enhancing factors	122 (2,318,500)	12.5 (12.0-13.0)	8.2 (7.9-8.5)	4.3 (4.1-4.5)	15.3 (14.6-15.9)	10.0 (9.6-10.4)	5.3 (5.0-5.5)
					290.2 (278.1-302.3)	190.1 (182.1-198.0)	100.1 (95.9-104.3)

Estimates combining strata may not total overall due to rounding error. Those of other races, n = 8 (85,948), are not shown.

Abbreviations as in Tables 1 and 2.

a Post-treatment values calculated by multiplying pretreatment values by 0.655.

**Table 4:** Estimated Cardiovascular Events and Preventable Events in Diabetes Population, Preinclisiran and Postinclisiran Treatment, Based on Pooled Cohort Equation ASCVD Risk Score

	n (N)	10-Year CVD Risk Pretreatment, % (95% CI)	10-Year CVD Risk Post-Treatment, % (95% CI)^a^	Difference, % (95% CI)	CVD Events Pre n (95% CI)	CVD Events Post n (95% CI)	Difference (Preventable Events) n (95% CI)
					K (95% CI)	K (95% CI)	K (95% CI)
Overall	516 (6,471,621)	24.5 (23.1-25.9)	17.4 (16.4-18.4)	7.1 (6.7-7.5)	126.4 (119.3-133.8)	89.8 (84.7-95.0)	36.7 (34.6-38.8)
					1,585.5 (1,496.6-1,678.1)	1,125.7 (1,062.6-1,191.5)	459.8 (434.0-486.7)
Females	275 (3,427,179)	21.4 (19.4-23.4)	15.2 (13.8-16.6)	6.2 (5.6-6.8)	58.9 (53.4-64.4)	41.8 (37.9-45.7)	17.1 (15.5-18.7)
					733.4 (665.9-802.6)	520.7 (472.8-569.8)	212.7 (193.1-232.7)
Males	241 (3,044,442)	28.0 (26.1-29.9)	19.9 (18.5-21.2)	8.1 (7.6-8.7)	67.5 (62.9-72.1)	47.9 (44.7-51.2)	19.6 (18.3-20.9)
					852.4 (795.1-911.1)	605.2 (564.5-646.9)	247.2 (230.6-264.2)
White	157 (3,981,183)	25.6 (22.9-28.3)	18.2 (16.2-20.1)	7.4 (6.6-8.2)	40.2 (35.9-44.4)	28.5 (25.5-31.5)	11.7 (10.4-12.9)
					1,019.2 (910.1-1,126.0)	723.6 (646.1-799.5)	295.6 (263.9-326.5)
Asian	56 (386,052)	23.9 (19.8-28.0)	17.0 (14.1-19.8)	6.9 (5.8-8.1)	13.4 (11.1-15.7)	9.5 (7.9-11.1)	3.9 (3.2-4.5)
					92.3 (76.6-107.9)	65.5 (54.4-76.6)	26.8 (22.2-31.3)
Black	154 (1,040,764)	25.7 (23.3-28.0)	18.2 (16.6-19.9)	7.5 (6.8-8.1)	39.6 (35.9-43.1)	28.1 (25.5-30.6)	11.5 (10.4-12.5)
					267.5 (242.9-291.2)	189.9 (172.5-206.7)	77.6 (70.4-84.4)
Hispanic	134 (890,704)	20.5 (18.0-22.9)	14.6 (12.8-16.2)	5.9 (5.2-6.6)	27.5 (24.2-30.7)	19.5 (17.2-21.8)	8.0 (7.0-8.9)
					182.6 (160.6-203.8)	129.6 (114.0-144.7)	53.0 (46.6-59.1)
Age <65 y	278 (3,848,688)	12.9 (12.0-13.8)	9.2 (8.5-9.8)	3.7 (3.5-4.0)	35.9 (33.3-38.5)	25.5 (23.7-27.3)	10.4 (9.7-11.2)
					497.0 (461.5-532.5)	352.8 (327.6-378.1)	144.1 (133.8-154.4)
Age ≥65 y	238 (2,622,932)	41.6 (39.7-43.4)	29.5 (28.2-30.8)	12.1 (11.5-12.6)	98.9 (94.5-103.4)	70.2 (67.1-73.4)	28.7 (27.4-30.0)
					1,090.4 (1,041.3-1,139.5)	774.2 (739.3-809.1)	316.2 (302.0-330.5)

Estimates combining strata may not total overall due to rounding error. Those of other races, n = 15 (172,917), are not shown.

Abbreviations as in Tables 1 and 2.

a Post-treatment values calculated by multiplying pretreatment values by 0.71.

In the secondary prevention population, overall pretreatment risk was 30.7% and was higher in males (31.7%) than in females (30.6%). Among race/ethnicity groups, Black patients had the highest risk (36.0%), and Asian patients had the lowest (24.8%). Patients aged ≥65 years had a much higher pretreatment risk (36.2%) compared with those aged <65 years (22.6%). From applying the projected inclisiran treatment effect of a 29.0% risk reduction as described previously, there was an overall absolute CVD risk reduction of 8.9%. An estimated ∼711,000 ASCVD events over 10 years were estimated to be preventable overall in the secondary prevention population. Preventable events were higher in males (∼457,000) than in females (∼266,400). Further noteworthy findings include ∼558,100 preventable events in White patients and ∼193,400/∼526,600 preventable events in those aged <65/≥65 years, respectively. Populations with the highest difference in 10-year CVD risk between pretreatment and post-treatment were Black patients (10.4%) and patients ≥65 years of age (10.5%). Most patients with ASCVD in our analysis were in the very high-risk group (5.9 million) compared with the not very high-risk group (2.1 million), and there were more preventable ASCVD events in the very high-risk group than in the not very high-risk group (∼540,200 and ∼182,900, respectively).

In the high-risk primary prevention population, pretreatment risk was 24.0% for the overall sample and was higher in females (25.5%) than in males (22.1%). Among race/ethnicity groups, in contrast with the secondary prevention population, Black patients had the lowest risk (19.3%), and Hispanic patients had the highest (27.1%). Patients aged ≥65 years once again had a much higher pretreatment risk (28.1%) compared with those aged <65 years (12.4%). After applying the projected inclisiran treatment effect of a 34.5% risk reduction as described previously, there was an overall absolute ASCVD risk reduction of 8.3%, resulting in ∼448,600 preventable ASCVD events over 10 years for the overall sample and ∼359,700 preventable events in White patients. Among race/ethnicity groups, Hispanic patients had the largest difference in 10-year CVD risk between pretreatment and post-treatment (9.3%). Preventable events were higher in females (∼260,500) compared with males (∼187,300), and preventable events were higher in those aged ≥65 years (∼389,100) compared with those aged <65 years (∼60,100). Finally, there were more patients in the primary prevention group with a 10-year risk score of ≥20% (3.1 million) compared with patients with a 10-year risk score of 7.5% to <20% with 2 or more risk-enhancing factors (2.3 million); preventable ASCVD events in these groups were ∼348,500 and ∼100,100, respectively.

In the diabetes population, pretreatment risk was 24.5% overall and was higher in males (28.0%) than in females (21.4%). Among race/ethnicity groups, White and Black patients had the similar risk (25.6% and 25.7%, respectively), and Hispanic patients had the lowest risk (20.5%). Patients aged ≥65 years had a much higher pretreatment risk (41.6%) compared with those aged <65 years (12.9%). After applying the projected inclisiran treatment effect of a 29.0% reduction in risk, the overall absolute ASCVD risk reduction was 7.1%, resulting in ∼459,800 preventable ASCVD events over 10 years for the overall sample and ∼295,600 preventable events in White patients. Preventable events were similar in males (∼247,200) and females (∼212,700), whereas preventable events were higher in those aged ≥65 years (∼316,200) compared with those aged <65 years (∼144,100).

## Discussion

Our study identified ∼20 million adults eligible for inclisiran across our primary and secondary prevention and diabetes populations with inadequately controlled LDL-C despite statin therapy. Based on expected risk reductions extrapolated from prior statin trials, we estimated that inclisiran treatment may prevent over 1.6 million ASCVD events over 10 years, including approximately 711,000 among secondary prevention, 448,600 among primary prevention, and 459,800 among diabetes patient populations. These findings highlight the potential benefits of inclisiran beyond statins alone when given to high-risk primary prevention, secondary prevention, and diabetes patient populations on statin therapy but at suboptimal LDL-C levels.

Although cardiovascular outcomes trials for inclisiran are ongoing both for pre-existing ASCVD and high-risk primary prevention patients, the therapy is currently indicated by the FDA as an adjunct to diet and exercise for the treatment of adults with hypercholesterolemia, to reduce LDL-C. The most recent ACC Expert Consensus Decision Pathway on nonstatin therapy^3^ considers the use of inclisiran as an option for ASCVD patients who despite maximally tolerated statin therapy still have LDL-C 70 mg/dL or higher, or if defined to be very high-risk ASCVD, 55 mg/dL or higher. Most recently, the 2025 ACC/AHA/ACEP/NAEMSP/SCAI Guideline for the Management of Patients With Acute Coronary Syndromes recommends nonstatin lipid-lowering therapy, such as inclisiran, to further reduce the risk of adverse cardiovascular outcomes for patients on maximally tolerated statins with LDL-C ≥70 mg/dL (and may be reasonable if LDL-C ≥55 mg/dL).^15^ Notably, in a recent pragmatic trial (VICTORION-INITIATE) U.S. patients (n = 450) were randomized to inclisiran first or usual care. Those included in the “inclisiran first” group were less likely to discontinue statins (6.0% vs 16.7%) and more likely to achieve LDL-C goals of <70 mg/dL (81.8% vs 22.2%) and <55 mg/dL (71.6% vs 8.9%) within the primary analysis time-frame of 330 days.^23^

Of great interest is the potential for inclisiran to reduce ASCVD events beyond statin therapy. Cardiovascular outcomes trials of inclisiran are ongoing both for patients with pre-existing ASCVD (VICTORION-2 PREVENT^9^ and ORION-4^10^) and high-risk primary prevention patients with ≥20% 10-year ASCVD risk or 7.5% to <20% 10-year ASCVD risk with 2 or more risk-enhancing factors (VICTORION-1 PREVENT).^11^ However, in the recently reported patient-level analysis of the completed phase III trials of inclisiran, a predetermined exploratory endpoint found a ∼25% relative risk reduction in major adverse cardiovascular events over 18 months from inclisiran treatment compared with placebo, with LDL-C reduced by 1.38 mmol/L.^12^ This compares favorably with the CTT meta-analyses that included 22 trials of statin therapy; among patients in those trials with a history of vascular disease (most similar to the above comparable phase III trial population reported from inclisiran), there was a 21% relative risk reduction in future major vascular events per mmol/L reduction in LDL-C.^12^ With the assumption that the LDL-C lowering effect from inclisiran is similar to that of statins, this would project to a 29.0% risk reduction based on a 1.38 mmol/L reduction in LDL-C from inclisiran.

A key strength of this study is the use of the NHANES database, which provides sample weighting allowing U.S. population estimation of inclisiran-eligible U.S. adults and preventable ASCVD events among the ethnically diverse U.S. population. Moreover, our main CVD risk factors, namely blood pressure and lipids, in addition to medication usage, are all measured variables in NHANES. Furthermore, NHANES allows for stratification of findings by race/ethnicity.

### Study Limitations

One limitation of our study is the use of risk scores to estimate preventable ASCVD events. Although the SMART2 risk score and PCE ASCVD risk scores were established for estimating ASCVD events, like most risk scores, their estimation of ASCVD risk may differ from actual ASCVD events observed in cardiovascular outcomes clinical trials, and they have been known to overestimate actual events.^24^ Moreover, if future cholesterol management guidelines adopt the PREVENT (Predicting Risk of Cardiovascular Disease EVENTs) risk score,^25^^,^^26^ they may define risk differently than if using the PCE. This may impact the eligibility and risk estimations in our study as they are based on high-risk primary prevention eligibility criteria defined by the PCE. There are also several assumptions made that may affect the validity of our results. We did not factor in potential variation in LDL-C lowering effect according to demographic subgroup, as this was beyond the scope of this analysis. We also assumed that the projected ASCVD risk reduction from inclisiran will be proportional to the risk reduction achieved from prior statin trials per increment (mmol/L) of LDL-C lowering, and that any different pleiotropic effects of inclisiran, if present, would not augment or attenuate this effect. Moreover, our estimates were made on the basis of extrapolating the relative risk reductions from the CTT collaboration statin trials, with a mean follow-up of approximately 5 years,^19^ to our projections of risk reduction over 10 years of treatment with inclisiran; it is uncertain whether the effect of LDL-C lowering with inclisiran on cardiovascular outcomes will be similar to that observed with statins from the CTT analysis. Importantly, the statin trials included in the CTT analysis, from which our assumed LDL-C-lowering effect on ASCVD outcomes was derived, were conducted with comparator groups of placebo, usual care, or less intensive statin. Therefore, it is unknown whether a similar degree of LDL-C lowering from inclisiran would have similar benefit on top of the current, more-stringent standards of care (eg, high-intensity statin) as are used in the VICTORION primary and secondary prevention trials.^9^, ^10^, ^11^ Furthermore, some factors considered in the SMART2 risk score, namely abdominal aortic aneurysm and peripheral artery disease, were absent from the NHANES cycles that formed the basis of our study. As such, our risk estimates for secondary prevention, which assume these conditions are absent, would likely be conservative. Since the completion of this study, an FDA label update in July 2025 removed the requirement for inclisiran to be used in combination with statins. Thus, as the populations included in this analysis are based on the more limited eligibility criteria of ongoing inclisiran clinical trials, the eligible patient population in the United States could be even larger, representing a potential direction for future research.

## Conclusions

In conclusion, our study suggests that ∼20 million adults already receiving statins may potentially be eligible for inclisiran across primary and secondary prevention and diabetes populations. Furthermore, based on our projections, the use of inclisiran on top of statin therapy has the potential to prevent >1.6 million ASCVD events over 10 years in the populations analyzed here. Although these findings require verification using real-world patient populations, the use of inclisiran alongside statins could have a significant impact on reducing health care resources and costs associated with CVD.

## Funding support and author disclosures

This study was supported by a contract from 10.13039/100008272Novartis Pharmaceuticals with the University of California, Irvine. Dr Wong has received research support through his institution from 10.13039/100002429Amgen, 10.13039/100004336Novartis Pharmaceuticals, 10.13039/501100004191Novo Nordisk, and 10.13039/100009857Regeneron; consultant fees from Amgen, New Amsterdam, Ionis, and Novartis; and speaker fees from Novartis. Dr Electricwala is an employee of Novartis. All other authors have reported that they have no relationships relevant to the contents of this paper to disclose.

## References

[1] Colantonio L.D., Rosenson R.S., Deng L. (2019). Adherence to statin therapy among US adults between 2007 and 2014. J Am Heart Assoc.

[2] Wong N.D., Zhao Y., Quek R.G.W. (2017). Residual atherosclerotic cardiovascular disease risk in statin-treated adults: the multi-ethnic study of atherosclerosis. J Clin Lipidol.

[3] Shen M., Aghajani N.A., Nasir K., Bhatt D.L., Khera R. (2022). Contemporary national patterns of eligibility and use of novel lipid-lowering therapies in the United States. J Am Heart Assoc.

[4] Lloyd-Jones D.M., Morris P.B., Ballantyne C.M. (2022). 2022 ACC expert consensus decision pathway on the role of nonstatin therapies for LDL-cholesterol lowering in the management of atherosclerotic cardiovascular disease risk: a report of the American college of cardiology solution set oversight committee. J Am Coll Cardiol.

[5] ElSayed N.A., Aleppo G., Aroda V.R. (2023). 10. Cardiovascular disease and risk management: standards of care in diabetes-2023. Diabetes Care.

[6] Koskinas K.C., Gencer B., Nanchen D. (2021). Eligibility for PCSK9 inhibitors based on the 2019 ESC/EAS and 2018 ACC/AHA guidelines. Eur J Prev Cardiol.

[7] Wong N.D., Fan W., Philip S., Granowitz C., Toth P.P. (2020). REDUCE-IT eligibility and preventable cardiovascular events in the US population (from the National Health and Nutrition Examination Survey [NHANES]). Am J Cardiol.

[8] Ray K.K., Wright R.S., Kallend D. (2020). Two phase 3 trials of inclisiran in patients with elevated LDL cholesterol. N Engl J Med.

[9] ClinicalTrials.gov (2021). https://clinicaltrials.gov/study/NCT05030428.

[10] ClinicalTrials.gov ORION-4: A Double-Blind Randomized Placebo-Controlled Trial Assessing The Effects of Inclisiran on Clinical Outcomes Among People With Atherosclerotic Cardiovascular Disease. https://clinicaltrials.gov/study/NCT03705234.

[11] ClinicalTrials.gov (2023). https://clinicaltrials.gov/study/NCT05739383.

[12] Ray K.K., Raal F.J., Kallend D.G. (2023). Inclisiran and cardiovascular events: a patient-level analysis of phase III trials. Eur Heart J.

[13] National Center for Health Statistics (2024). https://www.cdc.gov/nchs/nhanes/index.html.

[14] Grundy S.M., Stone N.J., Bailey A.L. (2019). 2018 AHA/ACC/AACVPR/AAPA/ABC/ACPM/ADA/AGS/APhA/ASPC/NLA/PCNA guideline on the management of blood cholesterol: a report of the American college of cardiology/American heart association task force on clinical practice guidelines. J Am Coll Cardiol.

[15] Rao S.V., O'Donoghue M.L., Ruel M. (2025). 2025 ACC/AHA/ACEP/NAEMSP/SCAI guideline for the management of patients with acute coronary syndromes: a report of the American college of cardiology/American heart association joint committee on clinical practice guidelines. Circulation.

[16] Andary R., Fan W., Wong N.D. (2019). Control of cardiovascular risk factors among US adults with type 2 diabetes with and without cardiovascular disease. Am J Cardiol.

[17] Hageman S.H.J., McKay A.J., Ueda P. (2022). Estimation of recurrent atherosclerotic cardiovascular event risk in patients with established cardiovascular disease: the updated SMART2 algorithm. Eur Heart J.

[18] Goff D.C., Lloyd-Jones D.M., Bennett G. (2014). 2013 ACC/AHA guideline on the assessment of cardiovascular risk: a report of the American college of cardiology/American heart association task force on practice guidelines. J Am Coll Cardiol.

[19] Fulcher J., O'Connell R., Voysey M. (2015). Efficacy and safety of LDL-lowering therapy among men and women: meta-analysis of individual data from 174,000 participants in 27 randomised trials. Lancet.

[20] Kearney P.M., Blackwell L., Collins R. (2008). Efficacy of cholesterol-lowering therapy in 18,686 people with diabetes in 14 randomised trials of statins: a meta-analysis. Lancet.

[21] Wong N.D., Young D., Zhao Y. (2016). Prevalence of the American college of cardiology/American heart association statin eligibility groups, statin use, and low-density lipoprotein cholesterol control in US adults using the national health and nutrition examination survey 2011-2012. J Clin Lipidol.

[22] Wong N.D., Karthikeyan H., Fan W. (2025). US population eligibility and estimated impact of semaglutide treatment on obesity prevalence and cardiovascular disease events. Cardiovasc Drugs Ther.

[23] Koren M.J., Rodriguez F., East C. (2024). An “inclisiran first” strategy vs usual care in patients with atherosclerotic cardiovascular disease. J Am Coll Cardiol.

[24] Rana J.S., Tabada G.H., Solomon M.D. (2016). Accuracy of the atherosclerotic cardiovascular risk equation in a large contemporary, multiethnic population. J Am Coll Cardiol.

[25] Khan S.S., Coresh J., Pencina M.J. (2023). Novel prediction equations for absolute risk assessment of total cardiovascular disease incorporating cardiovascular-kidney-metabolic health: a scientific statement from the American heart association. Circulation.

[26] Khan S.S., Matsushita K., Sang Y. (2024). Development and validation of the American heart association’s PREVENT equations. Circulation.

