# Supplementary material for: Potential Eligibility and Estimated Preventable Cardiovascular Disease Events From Inclisiran Treatment in the United States
Source: JACC Adv. 2026 Jan 20;5(2):102517. doi: 10.1016/j.jacadv.2025.102517 (PMC12859191; doi:10.1016/j.jacadv.2025.102517)
Supplement: Supplemental Material [file mmc1.pdf]

Supplemental Appendix:

SMART2 Model for Secondary Prevention Population:

LP_i= -0.03496022*(age, years) + 0.000551072*(age squared, years) - 0.21072103*(aspirin or equivalent, 1=yes, 0=no) + 0.294701954*(coronary artery disease, 1=yes, 0=no) - 0.03967521*(eGFR [CKDEPI], mL/min/1.73 m^2) + 0.000218613*(eGFR squared [CKDEPI] , mL/min/1.73 m^2) + 0.151760173*ln(hsCRP, mg/L) + 0.345583271*(current smoking, 1=yes, 0=no) + 0.34831786*(cerebrovascular disease, 1=yes, 0=no) + 0.318170659*(diabetes mellitus, 1=yes, 0=no) + 0.540364249*ln(non HDL-C, mg/dL) + 0.018913154*(systolic blood pressure, mmHg) + 0.287658743*(male sex, 1=yes, 0=no) + 0.047699585*(years since first ASCVD diagnosis) - 0.00164973*(years since first ASCVD diagnosis, squared)

$$CVDrisk_{10} = 1 - 0.870101479^{exp(LP_i - 4.7663518952)}$$

AHA/ACC Model for High-Risk Primary Prevention and Diabetes Populations:

For Black male patients:

LP_i= 2.469*ln(age, years) + 0.302*ln(total cholesterol, mg/dL) - 0.307*ln(HDL-C, mg/dL) + 1.916*ln(systolic blood pressure, mmHg) if treated for high blood pressure + 1.809*ln(systolic blood pressure, mmHg) if untreated for high blood pressure + 0.549*(current smoker, 1=yes, 0=no) + 0.645*(diabetes mellitus, 1=yes, 0=no)

$$CVDrisk_{10} = 1 - 0.8954^{exp(LP_i - 19.54)}$$

For other male patients:

LP_i= 12.344*ln(age, years) + 11.853*ln(total cholesterol, mg/dL) - 2.664*ln(total cholesterol, mg/dL)*ln(age, years) - 7.990*ln(HDL-C, mg/dL) + 1.769*ln(HDL-C, mg/dL)*ln(age, years) + 1.797*ln(systolic blood pressure, mmHg) if treated for high blood pressure + 1.764*ln(systolic blood pressure, mmHg) if untreated for high blood pressure + 7.837*(current smoker, 1=yes, 0=no) - 1.795*(current smoker, 1=yes, 0=no)*ln(age, years) + 0.658*(diabetes mellitus, 1=yes, 0=no)

$$CVDrisk_{10} = 1 - 0.9144^{exp(LP_i - 61.18)}$$

For Black female patients:

LP_i= 17.114*ln(age, years) + 0.940*ln(total cholesterol, mg/dL) - 18.920*ln(HDL-C, mg/dL) + 4.475*ln(HDL-C, mg/dL)*ln(age, years) + 29.291*ln(systolic blood pressure, mmHg) if treated for high blood pressure - 6.432*ln(systolic blood pressure, mmHg) if treated for high blood pressure*ln(age, years) + 27.820*ln(systolic blood pressure, mmHg) if untreated for high blood pressure - 6.087*ln(systolic blood pressure, mmHg) if untreated for high blood pressure*ln(age, years) + 0.691*(current smoker, 1=yes, 0=no) + 0.874*(diabetes mellitus, 1=yes, 0=no)

$$CVDrisk_{10} = 1 - 0.9533^{exp(LP_i - 86.61)}$$

For other female patients:

LP_i= -29.799*ln(age, years) + 4.884*ln(age, years)*ln(age, years) + 13.540*ln(total cholesterol, mg/dL - 3.114*ln(total cholesterol, mg/dL)*ln(age, years) - 13.578*ln(HDL-C, mg/dL) + 3.149*ln(HDL-C, mg/dL)*ln(age, years) + 2.019*ln(systolic blood pressure, mmHg) if treated for high blood pressure) + 1.957*ln(systolic blood pressure, mmHg) if untreated for high blood pressure + 7.574*(current smoker, 1=yes, 0=no) - 1.665*(current smoker, 1=yes, 0=no)*ln(age, years) + 0.661*(diabetes mellitus, 1=yes, 0=no)

$$CVDrisk_{10} = 1 - 0.9665^{exp(LP_i + 29.18)}$$

ACC = American College of Cardiology; AHA = American Heart Association; ASCVD = atherosclerotic cardiovascular disease; CKDEPI = The Chronic Kidney Disease Epidemiology Collaboration; CVD = cardiovascular disease; eGFR = epidermal growth factor receptor; HDL-C = high-density lipoprotein cholesterol; hsCRP = high-sensitivity C-reactive protein; SBP = systolic blood pressure.
